# Parliament's One‐Year Review of the Coronavirus Act 2020: Another Example of Parliament's Marginalisation in the Covid‐19 Pandemic

**DOI:** 10.1111/1467-923X.13048

**Published:** 2021-08-13

**Authors:** Daniella Lock, Pablo Grez Hidalgo, Fiona de Londras

**Keywords:** Coronavirus Act 2020, pandemic, Parliament, government accountability, legislative scrutiny, parliamentary review

## Abstract

In this article, we consider the one‐year review (OYR) by Parliament of temporary powers in the Coronavirus Act 2020 (CVA). The OYR stands as a key concession on the part of the UK government to enable scrutiny of Covid‐19 law making, after the CVA was rushed through Parliament at the beginning of the pandemic. The principal argument of this article is that despite appearances, this review was another example of Parliament being marginalised during the Covid‐19 pandemic. In particular, there were four obstacles to meaningful scrutiny in the OYR: inadequate parliamentary time scheduled for the review; the ‘all‐or‐nothing’ framing of the review; late and inaccurate government reporting prior to the OYR; and the failure to address key issues regarding the operation of the CVA, including major human rights concerns. In light of such obstruction to scrutiny, it is clear that the review represents a broken promise on the part of the current government to Parliament. The review is also part of a broader pattern of marginalising Parliament during the pandemic. In presenting this analysis, we argue that two changes could be made in the upcoming and penultimate review of the CVA in September 2021, in order to enable Parliament to engage in meaningful scrutiny in this review.

## Legislating in emergency mode: the Coronavirus Act 2020

AS WE EXPERIENCE another summer living with Covid‐19 and its variants, it is clear that managing the pandemic is likely to be part of daily life for some time to come. However, even as Covid‐19 is absorbed into ‘the new normal’, the government's response continues to be framed by an emergency paradigm. This is underpinned largely by the CVA, fast‐tracked through Parliament a few days before its recess in March 2020.

These powers enable the government to take control over vast areas of public life: to shut ports and gatherings; to close schools and nurseries; to postpone subnational elections and referendums; and to detain individuals under the Mental Health Acts with the permission of just one doctor rather than two. It also allows local authorities in England and Wales to reduce the availability of care and support to the extent that this does not result in a breach of an individual's rights under the European Convention on Human Rights (ECHR). When the CVA was going through Parliament, the Shadow Health Secretary, Jonathan Ashworth MP, described such powers as the ‘most draconian … ever seen in peacetime Britain’, and referred to the ‘huge potential for abuse’ of such powers ‘however well intended and needed’.^1^

As many parliamentarians observed when the CVA was being passed, the short time available for Parliament to scrutinise these powers before they became law was a cause for significant concern. In the House of Lords, Baroness Bennett (Greens) described the fast tracking of the bill as a ‘profoundly undemocratic, rushed but essential process’.^2^ In the House of Commons, Ian Blackford MP (Scottish National Party) stressed that the bill could not be scrutinised in the way that Parliament would ‘normally wish’, while emphasising that the ‘imposition of measures that will significantly alter individual liberties deserves full and frank scrutiny, no matter the context’.^3^ Sarah Jones MP (Labour) drew attention to the extremely short time MPs had to consider government amendments, such as key amendments on housing, which they had had only fifteen minutes to consider by the time of her speech.^4^

Even Conservative MPs highlighted the lack of time Parliament had to scrutinise the CVA. For example, in the House of Commons at the second reading, Jeremy Hunt MP noted that ‘[o]rdinarily, our role as MPs is to scrutinise every detail of legislation, to understand it and to try to improve it’ and noted there were ‘many questions’ about the CVA, while highlighting that delays could cost lives when the UK was facing a national emergency.^5^

## A constitutionally appropriate promise of parliamentary scrutiny…at a later date

In recognition of this short timeframe for debate, and as a concession on the part of the government, Parliament was told it would have further opportunities to subject the powers in the CVA to scrutiny in the form of six‐monthly reviews of the act. Before the second reading of the act in the House of Commons, a cross‐party amendment was tabled to review the CVA every six months and vote for its renewal. This amendment became section 98 of the CVA, which provides that the government must make arrangements for debate and a vote on the motion which states that ‘the temporary provisions of the Coronavirus Act 2020 should not yet expire’. If the House of Commons rejects that motion, the temporary provisions of the CVA expire ‘not later than the end of the period of 21 days from the time of the rejection’. Notably, the House of Lords has no role in this six‐monthly review process, something Lord Newby (Liberal Democrats) described as ‘unprecedented and completely unacceptable’.^6^

Matt Hancock MP, then the Health Secretary, described the six‐month review as an ‘additional safeguard’, claiming that it would enable meaningful periodic parliamentary scrutiny. In doing so, he assured Parliament that the government would ‘provide evidence and advice from the chief medical officer to inform the debate’ and reminded them that the act also included a ‘reporting mechanism for a report every eight weeks on the use of the powers in the Bill’ (contained in section 97 of the CVA).^7^

The government's promise of later parliamentary scrutiny purported to recognise Parliament's role within the UK constitutional order. However, numerous MPs noted that the mode of review suggested had significant limitations. First, a number of parliamentarians argued that six months was too long to wait for a review of this kind, asking what would happen if people experienced negative effects between reviews (Catherine West MP, Labour) and arguing that six months from the passage of the CVA was ‘simply too late for Parliament to have its first chance formally to decide whether this very wide‐ranging legislation should continue’ (Lord Newby, Liberal Democrats).^8^ Secondly, several MPs raised concerns about being unable to amend the act when reviewing it. For example, David Davis MP (Conservative) stated that in being forced to take an ‘all or nothing’ approach in the absence of an ability to amend the legislation, MPs would ‘perhaps be faced with eight good bits of legislation and one or two bits that are doing badly’ which would force them to ‘vote the whole thing through, rendering it a rubber stamp’.^9^

## The appearance of scrutiny

This short account shows that even when assured that the six‐monthly review would ensure Parliament could subject the CVA to scrutiny, many MPs were sceptical of whether such delayed scrutiny would be meaningful in practice. Such scepticism proved prescient.

The first such vote—six months after the passage of the CVA—did little to assuage MPs’ concerns. This was partly because it was scheduled for a paltry ninety minutes of debate, and partly because it coincided in time with the Brady amendment (insisting on parliamentary scrutiny of regulations under the Public Health (Control of Disease) Act 1984 (PHA), through which ‘lockdowns’ were introduced and shaped), which ended up dominating the debate even though it did not clearly relate to the CVA itself. However, by the time of the second six‐month review (that is, the OYR) those matters had largely been settled: the government had committed to ensuring Parliament could debate PHA regulations before they were introduced. Moreover, while the pandemic was still extremely challenging from a public health and governance perspective, there was a substantially greater degree of scientific, social and political understanding of its nature and effects than there had been when the CVA was introduced. There was also, by then, a greater intensity of public and political debate about the necessity, effectiveness, proportionality and sufficiency of measures introduced under both the CVA and the PHA to deal with the pandemic.

It is thus, perhaps, to be expected that to at least some degree the OYR appears, on the face of it, to have offered an opportunity for effective scrutiny. First, rather than what Charles Walker MP (Conservative) had called the ‘utter, utter disgrace’, of a ninety‐minute motion as in the six‐month review, the OYR was scheduled for 210 minutes in the House of Commons.^10^ Second, with more time and without the distraction of an internal Conservative Party dispute on parliamentary involvement—as had occurred in relation to the Brady Amendment in September 2020—MPs raised a number of human rights issues related to the operation of the CVA. These included: preventable deaths from coronavirus; inequalities in deaths from coronavirus; the use of Schedule 21 and powers to detain potentially infectious people; wrongful prosecutions; the treatment of individuals in care homes; the right to protest; the provision of healthcare to non‐coronavirus sufferers; mental health during the pandemic; disproportionate impact of the pandemic on BAME populations; and the potential need for a public inquiry into the government's handling of the pandemic. That MPs were able to present this range of human rights to the government in the debate points towards the review being one of substance.

Finally, it is notable that some of the more controversial aspects of the CVA had been expired by ministers under section 90 of the CVA prior to the OYR. This includes provisions that enabled local authorities to reduce social care (to the extent that this does not breach ECHR rights), and controversial provisions loosening requirements for the detention of individuals under the Mental Health Acts. The expiry of these provisions in anticipation of the OYR might suggest that it was considered to be more than a rubber‐stamping exercise. However, in spite of all of this, there are three features of the OYR that complicate this apparent narrative and point instead to the continued marginalisation of Parliament and to superficial government engagement with the process.

## Obstacles to meaningful scrutiny

### Inadequate time to debate

The first feature was a lack of parliamentary time for MPs to scrutinise the operation of the CVA. Even though, as already noted, the OYR was scheduled for 210 minutes (that is, more than three times the time allocated to the six‐month review), in reality this time was not all devoted to the review of the CVA. Instead, the motion for the OYR was debated in conjunction with four other motions which meant that the allotted time was shared between five motions. These included a motion on the ‘Health Protection (Coronavirus, Restrictions) (Steps) (England) Regulations 2021’, the controversial new regulations that would govern the next phase of lockdown and, among other things, introduced extensive restrictions on travelling abroad, including creating a criminal offence of merely travelling to a port or airport in anticipation of non‐compliant foreign travel. There was also an extensive motion on parliamentary proceedings during the pandemic, and on the continued operation of the hybrid Parliament. These are hardly merely procedural or simple motions and, in practice, combining them with the motion on the OYR both reduced the amount of time for the review debate *and* required Parliament to engage in three different kinds of scrutiny at the same time: retrospective (on the operation of the CVA), prospective (on the potential operation of the new regulations), and procedural (on the workings of Parliament). The ‘real’ time allocated was, in reality, far less than it appeared.

We contend that this technique of loading multiple motions with different implications into one debate can be read as at best a downgrading and, at worst, a cynical obstruction of the opportunity for scrutiny that Parliament was promised when the six‐monthly reviews were introduced into the CVA. As Sir Charles Walker MP (Conservative) put it, the CVA ‘fundamentally change[d] the relationship between the individual and the state’. It required detailed and focussed review, particularly if the six‐monthly review process was in fact going to fulfil its purported function of restoring Parliament's role as central to the legislative framework for government responses to the Covid‐19 pandemic, bearing in mind the plausible argument for urgency in March 2020 and the resultant cooperation of more or less all parties (and, indeed, the devolved administrations) in agreeing to and securing the passage of the act when first introduced.

### A false dichotomy between ‘all or nothing’

A second obstacle to meaningful parliamentary scrutiny in the OYR (and, indeed, the six‐month review that preceded it) is its ‘all or nothing’ framing. This framing is produced through two techniques: the wording of the motion as provided for by the CVA, and the Health Secretary's presentation of the choice before the House as ‘all or nothing’.

As already mentioned, the motion that the House of Commons is invited to consider during the six‐month review debates is ‘[t]hat the temporary provisions of the Coronavirus Act 2020 should not yet expire’. In other words, the House of Commons is given the opportunity either to expire all or none of whatever temporary powers under the CVA are in operation at the time of the motion. There is no opportunity for them to vote that some ought to remain in force, and some be expired; their choice is a simple and binary one—all or nothing. As discussed above, at the time the CVA was being introduced, numerous MPs argued that this was insufficient to enable meaningful scrutiny by Parliament, and it is worth reiterating that point. Confronting the House of Commons with an opportunity merely to approve the continuation of all temporary provisions or none is almost akin to presenting it with no opportunity at all. For if even *one* of those provisions is considered to be necessary and effective in suppressing the coronavirus and/or mitigating its societal or economic effects, it would be extremely difficult for Parliament to vote *all* of the temporary provisions down, seeming to ‘sacrifice’ that of which it approves.

This challenge is exacerbated by the way in which the then Health Secretary presented the choice before the House of Commons in the OYR. He stated:

If we were to remove the temporary provisions in the Act altogether, we would lose, for instance, measures protecting commercial tenants and renters from eviction, we would not be able to run virtual court hearings, which are an integral part of maintaining the rule of law, and people would not be able to receive statutory sick pay for the full period for which they are required to self‐isolate.^11^

This statement reinforces the notion that voting against the motion would have serious negative effects—not only on the government's handling of the pandemic, but also for parts of public administration (such as court hearings) vital to everyday life, and for valued protections for individuals like sick pay. In other words, it frames the choice before the House of Commons not as a choice to require the government to expire the temporary provisions within twenty‐one days of the vote (as the statute provides), giving it enough time to introduce new provisions maintaining that which the House of Commons had indicated it approved of as valuable interventions in the pandemic response. Rather, the framing presents the vote as a nuclear option which any politician would be reluctant to take. Dawn Butler MP (Labour) picked up on this in the OYR, underlining that a vote against the motion would trigger this twenty‐one‐day period in which to pass a new act, containing any powers MPs wanted to keep from the CVA. Indeed, Butler tabled a bill that could replace the CVA in full, or from which replacement powers could be drawn if the twenty‐one‐day period was triggered by a negative vote. Her bill was not allocated any time and while it was referred to, in the abstract, a handful of times, none of the provisions contained in the bill were mentioned specifically or subject to debate.

### Late and inaccurate government reporting

Finally, it is notable that there were problems with the government's reporting in its annual report on the operation of the CVA, prior to the OYR taking place. This report, the sixth of the two‐month reports required under the CVA, is clearly intended to inform parliamentary scrutiny. However, the sixty‐eight page, often technical, report was not published until 22 March 2021, three days before the OYR, meaning MPs had little time to digest it.

The second, more serious, problem with the report was that it contained inaccurate information, with crucial implications for the OYR. The report stated that regulations made under section 24 of the CVA would be expired. This section provides the government with powers to extend time limits on the retention of fingerprints and DNA profiles being held in the interest of national security. This commitment to expire the regulations was welcomed in the OYR, with Steve Brine MP (Conservative) stating: ‘I have been through the one‐year report on the provisions of the Act … I note and welcome the parts that Ministers are retiring, such as … section 24, which gives the state crazy provision to retain the fingerprints and DNA profiles of my constituents.’^12^

The government did not respond to this statement, and the relevant provisions received no further consideration in the OYR, presumably because the report had asserted they would be expired. However, on 19 April 2021, during Parliament's recess, the government published a document of corrections to the report online.^13^ The corrections stated that, contrary to what the original report had claimed, only one of two regulations made under section 24 of the CVA would actually be expired. This meant that the Coronavirus (Retention of Fingerprints and DNA Profiles in the Interests of National Security) (No. 2) Regulations 2020 would continue to operate. As a result of this omission, Parliament had inaccurate information coming to the OYR and was deprived of the opportunity to raise critical questions regarding the operation of these extensive powers. Such questions included which safeguards have been put in place to ensure that the collection of data is limited to that which is strictly necessary for national security purposes. With the government having corrected the report during recess, it not only denied Parliament a crucial opportunity for scrutiny, but it did so without any real political consequences.

### The opportunity missed

We have already outlined that, despite assurances to the contrary, the government's engagement with the form and presentation of the OYR suggests that it was not committed to facilitating meaningful scrutiny, notwithstanding its assurances to the contrary. However, it is only right to acknowledge that in the limited time it *did* have available, the House of Commons missed the opportunity to ensure that it engaged substantively and rigorously with the effects and operation of the CVA. We want particularly to draw attention here to the failure to ensure robust engagement with the human rights implications of the act, and to draw from other processes—including inquiries by parliamentary committees—to ensure the integration of appropriate evidence and enhanced parliamentary scrutiny.

The CVA and, indeed, the whole government response to the pandemic has potentially serious and complex implications for human rights. On the one hand, there is a human rights obligation to take steps to protect life, to ensure health goods, services and facilities are available, accessible, acceptable and of good quality, and so on. On the other hand, the CVA and other powers introduced during the pandemic are in tension with rights to privacy, to equality and non‐discrimination, to family life, to fair trial, and more. It is very clear that the implications for rights of the pandemic are challenging and require close consideration as a matter both of law and political accountability. While human rights law does allow for limitations on many rights in pursuit of public health, these limitations must be proportionate, legal and necessary in a democratic society (as the ECHR test classically puts it). These tests are not static: what was proportionate or necessary in March 2020 may not have been so in March 2021, and while Parliament may not have had time to subject the CVA to robust human rights analysis in March 2020, one might expect such scrutiny a year later, when the implications of the CVA for rights had begun to become clear.

Notwithstanding this, engagement with rights during the OYR was suboptimal. We have already recognised that MPs raised some rights‐related issues that had previously received insufficient attention. However, even then, the engagement with rights is characterised by vagueness, imprecision and inadequacy. For example, William Wragg MP (Conservative) emphasised that ‘rights and freedoms are not in the ownership of the state, but are innate’, without mentioning any specific rights issues in relation to the CVA. Rachel Hopkins MP (Labour) stated that the government's handling of the pandemic ‘exacerbate[d] the inequalities in our society and … impact[ed] black, Asian and minority ethnic communities and disabled people disproportionately’.^14^ This statement was also made without reference to any specific information or recommendation.

More striking still is what was never raised by MPs in the OYR debate. For example, a key issue not mentioned was the human rights implications of the CVA changes to court proceedings, in relation to which the Joint Committee on Human Rights (JCHR) has raised several concerns.^15^ These include that access to justice has been undermined by the reliance on video link proceedings, and the potential exclusion of those who are vulnerable or without access to the relevant technology. Further concerns were raised that virtual courts were increasing how long some people were detained, and that children awaiting trial and who have been effectively serving time in prison without a sentence because of delays linked to the current system are particularly impacted.

A second key issue not mentioned was the unequal impact school closures were having on children across the UK, beyond a lack of access to education for those with special educational needs. Powers to close schools are contained in sections 37 and 38 in the CVA. The JCHR has highlighted that closures during the pandemic have resulted in ‘unequal access to education for disadvantaged children’.^16^ This included there being significant barriers to home learning for disadvantaged children, including poor internet access, insufficient access to devices or study spaces, and limited or no parental support. Despite the JCHR's findings, which were suggestive of a systemic undermining of the right to education in the UK, the widespread issue of access to education was not raised in the OYR.

In addition to missing key human rights issues, the OYR debate was also characterised by a failure to engage with crucial evidence. This includes a lack of reference by MPs to the two‐monthly reports on the CVA published under section 97 of the CVA, and the one year report on the act, which received only a very brief reference.^17^ Furthermore, only a handful references were made to reports by parliamentary committees, which have produced a vast amount of analysis and evidence over the course of the pandemic. These include the Women and Equalities Committee's report on the unequal impact of coronavirus and the government response to BAME people, the Constitution Committee's report on the pandemic and the role of Parliament, and a wealth of reports by the Public Accounts Committee on the government's provision of adequate healthcare during the pandemic (that is, the core objective of the CVA). Moreover, there were no references to important reports produced outside Westminster, such as the extensive work done by the National Audit Office, the Equality and Human Rights Commission, the Office of National Statistics, or indeed by NGOs working on the pandemic. This is notwithstanding the fact that independent research by all these organisations has highlighted crucial impacts of the Covid‐19 pandemic.

## Conclusions

This analysis of the OYR identifies two different sets of challenges to effective parliamentary scrutiny of the CVA: on the one hand the government's treatment of the six‐monthly motions as a procedural and substantive matter, and on the other hand, MPs’ failure to engage meaningfully with the substantive issues raised by the CVA, and to separate them out from general problems posed by the pandemic, from issues arising from regulations under the PHA, and from broader political disputes about Parliament's involvement in the pandemic. That the House of Commons undertook this debate with almost no reference whatsoever to important work offering MPs evidence of the (positive and negative) effects and operation of the act might partly be a product of the very short time allocated to the debate and the multiplicity of motions, but must also be recognised as a self‐imposed limitation on effective scrutiny by parliamentarians themselves.

That said, however, the government's treatment of the OYR motion is indicative of a wider pattern of parliamentary exclusion throughout the pandemic. This is particularly well illustrated by the fact that most of the provisions, limitations, restrictions and powers that people experience in their everyday lives do not emanate from the CVA at all, but rather from regulations made with extremely limited parliamentary involvement under section 45r of the PHA. There is a danger, though, in conceding to the government's continued marginalisation of Parliament through its cynical engagement with these six‐monthly reviews. It is easy—and indeed common in some other governance areas such as counter‐terrorism—for the government to claim to ‘compensate’ for a lack of scrutiny at the time of passing an act by offering *ex post facto* review of this kind, but for that claim to be credible, the postponed scrutiny *must* be meaningful and effective. There is thus much at stake—not only for the CVA, but also for Parliament as a constitutional and political actor—in the six‐monthly reviews of the CVA that remain.

The next opportunity for Parliament to scrutinise the CVA will be the penultimate review this September. This is likely to be the most substantive review, as the CVA should expire in its entirety in March 2022. To this end, we suggest two changes that might be made to make these reviews more effective and meaningful. First, adequate time must be given to the debate on the motion *and* the motion on the temporary powers under the CVA must be considered as a stand‐alone motion. This is particularly important because, as the planned duration of the CVA comes closer to an end, it is likely that government will begin to consider what features of the CVA might be considered to have been particularly efficient, effective, or desirable and begin to consider shifting them into permanent legislation so that they can be maintained ‘post‐pandemic’. The movement of provisions, approaches and powers from temporary into permanent legislation is a recognised phenomenon, and certainly there are some CVA features we can reasonably expect the government to want to maintain. For example, the government has already signalled its desire to maintain the provisions relating to the courts, and the expansion of live links for criminal proceedings has been included in the Police, Crime, Sentencing and Courts Bill.^18^

Secondly, at some point prior to the next review there should be an attempt to amend section 98 of the CVA so that the motion debated on a six‐month basis allows for some temporary provisions to be maintained and others to be expired by vote of the House of Commons, that is, to move away from the all or nothing approach currently adopted in the CVA. Indeed, David Davis MP (Conservative) proposed just such an amendment when the CVA was being debated in March 2020, so there is no complexity of drafting that would preclude such a move.^19^ The barriers to such an amendment are likely to be political (particularly within the Conservative Party) and pragmatic, with government likely arguing that since the CVA is due to expire in March 2022, such an amendment is unnecessary. However, we know that legislation like the CVA has a long afterlife; such statutes reflect previous legislative models and they, in turn, become the model for analogous legislation in the future. For Parliament to insist on a more consequential review power in the CVA would send an important message about future legislation. It would also send a crucial signal regarding its insistence on sustained institutional centrality and resistance to attempts to marginalise Parliament and undermine its function as an accountability forum within the UK's constitutional order.

## Notes

^1^

*House of Commons Debates*, 23 March 2020. vol. 674, col. 59.

^2^
HC Deb, 25 March 2020, vol. 802, col. 1769.

^3^
HC Deb, 23 March 2020, vol. 674, col. 70.

^4^
HC Deb, 23 March 2020, vol. 674, col. 93.

^5^
HC Deb, 23 March 2020, vol. 674, col. 61.

^6^
HL Deb, 25 March 2020, vol. 802, col. 1764.

^7^
HC Deb, 23 March 2020, vol. 674, cols. 36–37

^8^
HC Deb, 23 March 2020, vol. 674, col. 38 and vol. 802, col. 1764.

^9^
HL Deb, 23 March 2020, vol. 674, col. 70.

^10^
HC Deb, 23 March 2020, vol. 681, col. 411.

^11^
HC Deb, 25 March 2021, vol. 691, col. 1114.

^12^
HC Deb, 25 March 2021, vol. 691, col. 1137.

^13^
Corrections made to the One Year Report on the status on the non‐devolved provisions of the Coronavirus Act 2020, 19 April 2021; https://assets.publishing.service.gov.uk/government/uploads/system/uploads/attachment_data/file/978843/corrections‐to‐coronavirus‐act‐one‐year‐status‐report.pdf (accessed 3 August 2021).

^14^
HC Deb 25 March 2021, vol. 691, col. 1146.

^15^
Joint Committee on Human Rights, *The Government*'*s Response to COVID‐19* (2019–21, HC 265, HL 125) paras. 185–98.

^16^
Ibid, para. 178.

^17^
HC Deb 25 March 2021, vol. 691, col. 1137.

^18^
HC Deb 25 March 2021, vol. 691, col. 1116.

^19^
‘Committee of the Whole House Proceedings: Coronavirus Bill’ (25 March 2020) Supplement to the Votes and Proceedings, Not called 3.

